# Merkel cell carcinoma-derived exosome-shuttle miR-375 induces fibroblast polarization by inhibition of RBPJ and p53

**DOI:** 10.1038/s41388-020-01576-6

**Published:** 2020-12-11

**Authors:** Kaiji Fan, Ivelina Spassova, Jan Gravemeyer, Cathrin Ritter, Kai Horny, Anja Lange, Thilo Gambichler, Niels Ødum, David Schrama, Dirk Schadendorf, Selma Ugurel, Jürgen C. Becker

**Affiliations:** 1 Department of Translational Skin Cancer Research, University Hospital Essen, Essen, Germany; 2 German Cancer Consortium (DKTK), Essen, Germany; 3 German Cancer Research Center (DKFZ), Heidelberg, Germany; 4 Department of Dermatology, Medical University of Graz, Graz, Austria; 5 Research Group Bioinformatics, Faculty of Biology, University of Duisburg-Essen, Essen, Germany; 6 Skin Cancer Center, Department of Dermatology, Ruhr-University Bochum, Bochum, Germany; 7 LEO Foundation Skin Immunology Research Center, Department of Immunology and Microbiology, University of Copenhagen, Copenhagen, Denmark; 8 Department of Dermatology, University Hospital Würzburg, Würzburg, Germany; 9 Department of Dermatology, University Hospital Essen, Essen, Germany

**Keywords:** Cancer microenvironment, Skin cancer

## Abstract

Merkel cell carcinoma (MCC) is a highly invasive and metastatic skin cancer. While high expression of miR-375 is a characteristic of MCC, it seems not to contribute to the malignant phenotype of MCC cells. miR-375 enrichment in MCC-derived extracellular vesicles suggests its intercellular signaling function. Here, we demonstrate that horizontally transferred miR-375 causes fibroblast polarization toward cancer-associated fibroblasts (CAFs). The polarization is evidenced by phenotypic changes and induction of *α-SMA*, *CXCL2*, and *IL-1β*. Fibroblast polarization is inhibited by specific antagomirs and mimicked by experimental miR-375 expression. Mechanistically, miR-375 downregulates *RBPJ* and *p53*, two key players regulating fibroblast polarization. In clinical MCC samples, in situ hybridization located miR-375 in CAFs, which correlated with high α-SMA protein and low *RBPJ* and *TP53* expression; single-cell RNAseq revealed a disparate fibroblast polarization negatively correlating with p53 pathway-related gene expression. Thus, the functional role of miR-375 in MCC is to generate a pro-tumorigenic microenvironment by inducing fibroblast polarization.

## Introduction

Merkel cell carcinoma (MCC) is an aggressive neuroendocrine (NE) skin cancer with an estimated mortality rate of ~40% [1]. In most cases, MCC carcinogenesis is associated with the oncogenic Merkel cell polyomavirus (MCPyV), while in the other cases, it is UV-driven [1]. MCCs are heterogeneous tumors that are often infiltrated by various immune and stromal cells [2]. While the role of tumor-infiltrating lymphocytes has been studied in detail [3–5], the characteristics of tumor-associated fibroblasts in the MCC microenvironment are largely unexplored. Fibroblasts are very plastic cells that may contribute to tumor progression and metastasis depending on their polarization and activation states [6]. Hence, tumor-promoting fibroblasts are also termed cancer-associated fibroblasts (CAFs). CAFs are characterized by a spindle shape with elongated cytoplasmic processes [7], the expression of markers such as alpha-smooth muscle actin (α-SMA) [7, 8] and upregulation of pro-inflammatory genes such as C-X-C motif chemokine 2 (CXCL2) and interleukin 1 beta (IL-1β) [9, 10]. Fibroblast polarization is triggered via intrinsic and extrinsic mediators; however, this process is not fully understood [6]. Nevertheless, cancer cell-derived stimuli are assumed to be the major factors inducing CAF polarization. Their effect can be conveyed via direct cell-cell contact [11] or via soluble factors [12] or molecules that are mainly transported within extracellular vesicles (EVs), such as apoptotic blebs, microvesicles, or exosomes [13]. Exosomes are 30–130 nm in diameter, are actively released by the fusion of multivesicular endosomes with the plasma membrane and can stably persist in blood, lymph, and other bodily fluids [14]. Exosomes can contain a plethora of functional molecules, including proteins, DNAs, mRNAs, and non-coding RNAs. Thus, exosomes are important for endocrine and paracrine cell-cell communication [15]. microRNAs (miRNAs) are particularly common in exosomes because of their small size of 19-24 nucleotides. Notably, some miRNAs are selectively enriched in exosomes [16]; these miRNAs were classified as exosome-shuttle miRNAs [17]. The intercommunications between tumor and stromal cells via exosome-shuttle miRNAs impacts on tumor proliferation [18], angiogenesis [19, 20], metastasis [21, 22], immunity [23], as well as CAF activation [24, 25]. It should be noted, that the distinction of exosomes from other EVs is not trivial; thus, to be prudent we rather use the term EVs within this manuscript.

One of the most abundant miRNAs in MCC cells is miR-375 [26–29]. Its function in MCC has not yet been completely characterized, but it might promote NE differentiation of MCC cells [26]. Overexpression of truncated versions of the MCPyV-encoded large T antigen or the NE linage transcription factor ATOH1 induces miR-375 expression [30]. In addition to being expressed in MCC, miR-375 is expressed in medullary thyroid carcinoma [31], prostate cancer [32] and NE lung cancers [33]. The physiological function of miR-375 is best described by its role in pancreatic beta cells, in which it is an important regulator of insulin secretion [34]. miRNAs function by interfering with the stability of mRNA or inhibiting its translation; experimentally confirmed target genes of miR-375 are recombination signal binding protein for immunoglobulin kappa J region (*RBPJ*) and *TP53* [26, 35]. In most cancers, miR-375 has been deemed a tumor suppressor [36], but it also functions as an oncogenic miRNA depending on the cellular context [32, 33]. We recently reported that knockdown of miR-375 in MCC cell lines has—at best—minimal effects on cell survival, proliferation and morphology [37]. Highly efficient knockdown did not alter any of the signaling pathways involving miR-375 target genes [37], thus posing the question about the functional role of this highly abundant miRNA in MCC. Based on our observation that miR-375 is present in MCC conditioned cell culture medium as well as sera of MCC patients [27], it may function as an exosome-shuttle miRNA. Here, we provide evidence that horizontally transferred miR-375 is important for the polarization of fibroblasts toward a CAF phenotype in MCC.

## Results

### Fibroblasts in MCC tumors exhibit a CAF-like phenotype

**Fig. 1:**
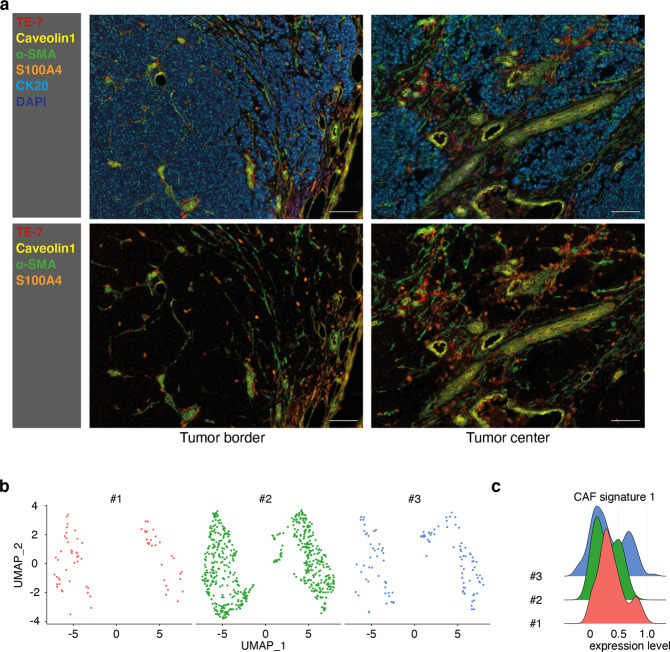
Fibroblasts in MCC tumors exhibit a CAF-like phenotype. **a** FFPE sections of MCC tissues were stained for the fibroblast markers TE-7 (red), Caveolin1 (yellow), α-SMA (green), and S100A4 (orange), as well as for the MCC tumor marker CK20 (light blue). Nuclei were stained with DAPI (dark blue). A representative MCC tissue is depicted. The scale bar represents 50 µm. **b** Fibroblasts visualized in a UMAP plot. Cells were annotated by mRNA expression profiles for known marker genes in three MCC tumors. **c** A CAF signature score was generated for fibroblasts in three tumors based on the mRNA expression of selected CAF marker genes (details are given in the “Material and Methods” section).

Based on our previous results [27, 37], we suggested that miR-375 acts in the intercellular signaling process via exosomal shuttling and can polarize stromal cells in the tumor microenvironment. Thus, we characterized fibroblasts in the MCC microenvironment by evaluating the expression of other fibroblast markers such as TE-7, Caveolin-1 (CAV1), α-SMA, and S100A4, using multiplex immunohistochemistry (mIHC) staining (*n* = 10, Supplementary Tables S1 and S2, example results shown in Fig. 1a and Supplementary Fig. S1). TE-7 was expressed at low levels in most stromal cells, with a few exceptions. α-SMA was expressed by fibroblast-like cells and pericytes in the vicinity of blood vessels and was often colocalized with CAV1. Unlike α-SMA, S100A4 was expressed mainly in single cells distributed throughout the tumor, with only some degree of co-localization with α-SMA. Notably, we did not observe obvious differences with respect to the fibroblast polarization depending on the presence or absence of MCPyV in the tumor. These morphological analyses demonstrate that MCC-associated fibroblasts are heterogeneous, although most exhibit a CAF-like phenotype.

Next, we performed single-cell RNA sequencing (scRNAseq) of three MCC tumors using 10x Genomics Chromium. T-distributed stochastic neighbor embedding (tSNE) clustered these cells into MCC tumor cells, fibroblasts, T lymphocytes, and endothelial cells (data not shown). Notably, tSNE separated the MCC-associated fibroblasts into two clusters based on their expression profiles in all three tumors (Fig. 1b). Most of the fibroblasts expressed well-established CAF markers, such as fibroblast activation protein; Thy-1 cell surface antigen; smooth muscle actin alpha 2 (α-SMA encoded by *ACTA2*); Caveolin 1 (CAV1); and different collagenases. However, these markers were expressed at different levels among the fibroblasts (Supplementary Fig. S2a). Thus, we generated a CAF score based on the expression profile of 88 CAF-associated genes that also identified two clusters of fibroblasts in all three tumors (Fig. 1c, Supplementary Table S3) [38]. In addition, combined fibroblast clusters from three tumors identified above were also classified into CAF^low^ and CAF^high^ groups according to their respective CAF score values (Supplementary Fig. S2b, c). To determine whether these two groups of fibroblasts in each tumor were independently developed or just at different stages of CAF polarization, we calculated the RNA velocity, which predicts the future state of individual cells on a time scale of hours based on the relative ratio of spliced and unspliced transcripts [39]. This analysis revealed that these two fibroblast clusters in all three tumors were directed toward different future states, as indicated by the RNA velocity vector visualized in by a principal component analysis (PCA) plot (Supplementary Fig. S2d). In summary, MCC-associated fibroblasts showed substantial heterogeneity and different polarization states, as revealed by the results of both immunohistochemistry-based staining and scRNAseq. On that basis, we next investigated whether these effects are triggered by horizontal transfer of miR-375.

### miR-375 is enriched in MCC-derived EVs and transferred into fibroblasts

**Fig. 2:**
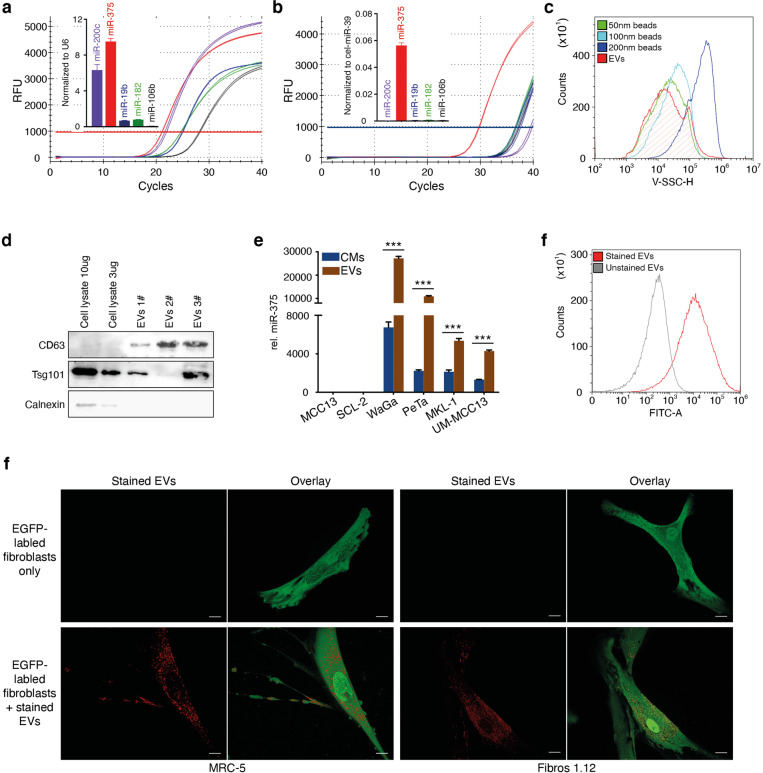
Fibroblasts take up MCC-derived EVs containing miR-375. The expression of miR-375, miR-182, miR-106b, miR-19b and miR-200c in WaGa cells (**a**) and CM from WaGa cells (**b**) was determined by RT-qPCR. The amplification curves and normalized relative expression level of each miRNA are shown. **c** EVs were isolated from WaGa CM. The sizes were determined by comparison to nanobeads of a defined size (50, 100, and 200 nm). **d** Expression of CD63, Tsg101 and Calnexin in WaGa cells and EVs derived from WaGa CMs were determined via immunoblot. **e** Relative miR-375 expression in EVs derived from four classical MCC cell lines (WaGa, PeTa, MKL-1, UM-MCC13) and a variant MCC cell line (MCC13) and SCL-2 cutaneous squamous cell carcinoma cells served as the control. Cq values were normalized to spiked-in cel-miR-39. **f** EVs isolated from WaGa cells were stained with Exo-Red; unstained EVs served as negative controls. **g** EGFP-expressing MRC-5 cells (left panels) or primary skin fibroblasts (Fibro.1.12, right panels) were cultured alone or in the presence of Exo-Red-labeled WaGa cell-derived EVs. Representative overlay images are shown; the scale bar represents 10 µm. Experiments were biologically replicated trice and were performed in triplicates. The error bars indicate the SDs; *** indicates *p* < 0.001.

We confirmed the abundance of miR-375 and its function as an exosome-shuttle miRNA in MCC cell lines by using quantitative real-time polymerase chain reaction (RT-qPCR) to compare the abundances of previously reported highly expressed miRNAs (miR-19b, miR-106b, miR-375, miR-200c, and miR-182) in WaGa cells and in conditioned medium (CM) from WaGa cells (Fig. 2a, b) [27, 28]. While all tested miRNAs were abundant in the cell lysate, only miR-375 was present in relevant amounts in the CM. The strong predominance of miR-375 in the cell-free supernatant suggests the active and selective release of miR-375 by WaGa cells, presumably via exosomal enrichment [40]. To test this hypothesis, we isolated EVs from the CM of classical miR-375-expressing MCC cell lines (WaGa, PeTa, MKL-1, and UM-MCC-13); these EVs were typically sized between 50 and 100 nm (Fig. 2c), and positive for CD63 and Tsg101, two common EV markers, and negative for Calnexin, a marker for the endoplasmic reticulum as identified by immunoblot (Fig. 2d). miR-375 was strongly enriched in these EVs compared to the corresponding CM but it could not be detected in EVs derived from the miR-375-negative cell lines MCC13 and SCL-2 (Fig. 2e). The MCC-derived EVs were stained with Exo-Red to investigate whether they could be taken up by fibroblasts (Fig. 2f). MRC-5 fibroblasts and Fibro1.12 primary skin fibroblasts uniformly incorporated the labeled EVs within 3 h (Fig. 2g). These observations strongly support the hypothesis that horizontal transfer of miR-375 from MCC cells to fibroblasts contributes to intercellular communication.

### MCC-derived factors polarize fibroblasts toward a CAF phenotype

**Fig. 3:**
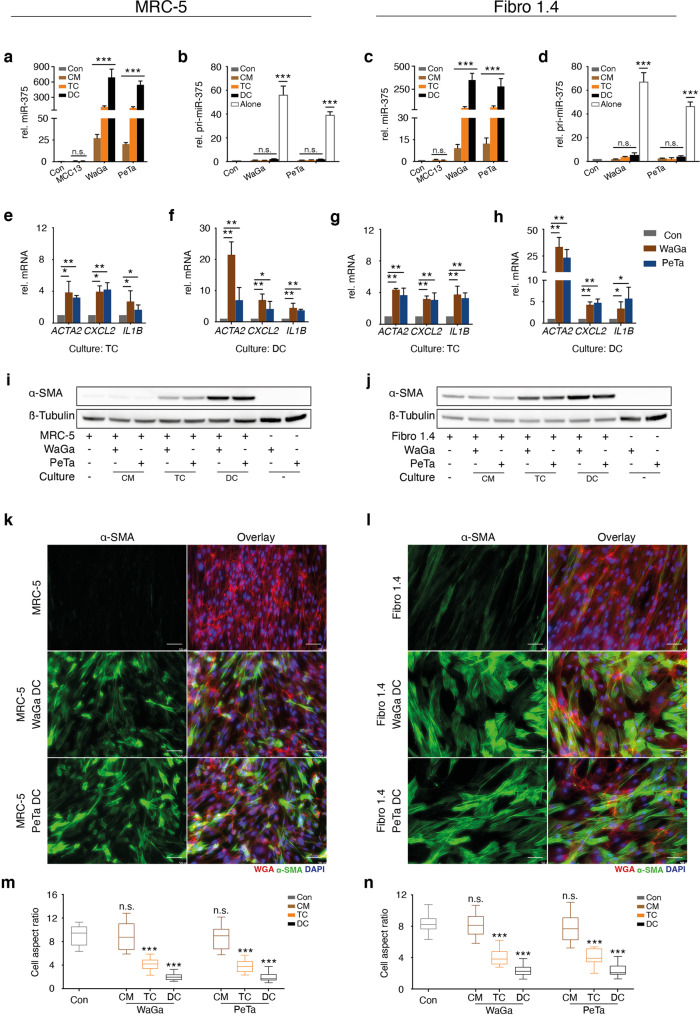
miR-375 is horizontally transferred from MCC cells to fibroblasts and induces a CAF-like phenotype. MRC-5 cells (**a, b, e, f, i, k, m**) or primary skin fibroblasts (Fibro1.4) (**c, d, g, h, j, l, n**) were cultured in MCC conditioned medium (CM), in a Transwell chamber system (TC) or in direct cell-cell contact (DC) with MCC13, WaGa or PeTa cells. **a- d** The relative expression levels of miR-375 (**a, c**) and pri-miR-375 (**b, d**) in fibroblasts were determined by RT-qPCR. **e–h** The relative *ACTA2*, *CXCL-2*, and *IL1B* mRNA expression levels were determined in fibroblasts under TC conditions (**e, g**) or DC conditions (**f, h**) by RT-qPCR. **i, j** α-SMA protein expression in fibroblasts cocultured with MCC cells was determined by immunoblotting; β-tubulin served as loading control. IF staining of α-SMA (green) in MRC-5 (**k**) and Fibro1.4 cells (**l**) after direct coculture with MCC cells. Cellular membranes were stained with WGA (red); nuclei, with DAPI (blue). The graphs show the values of cell aspect ratio (length/ width) of MRC-5 (**m**) and Fibro1.4 (**n**) under indicated conditions (*n* = 50), presented as box-and-whisker plots. The scale bars represent 50 µm. For RT-qPCR experiments, Cq values were normalized to U6 or *HPRT* expression and compared to the ΔCq value of untreated MRC-5 or Fibro1.4 cells, respectively. Experiments were biologically replicated trice and were performed in triplicates. The error bars indicate the SDs; * indicates *p* < 0.05, ** indicates *p* < 0.01, *** indicates *p* < 0.001.

To explore the functional impact of MCC-derived factors on fibroblasts, we performed a series of experiments co-culturing miR-375 expressing WaGa, PeTa cells, or miR-375-negative MCC13 cells with fibroblasts. Three different coculture conditions were tested: with direct cell-cell contact (DC), in a Transwell chamber system (TC) and in MCC CM. For all subsequent experiments, we used both the fibroblast cell line MRC-5 and primary fibroblasts. Although primary skin fibroblasts are biologically more relevant, they are characterized by a limited expansion capacity; thus, primary skin fibroblasts from different donors had to be used to ensure both reproducibility and biological relevance. Due to the different growth patterns of fibroblasts (adherent) and classical MCC cells (in suspension), they can be easily separated before subsequent analysis. However, the variant MCC cell line MCC13 does not exhibit the classical MCC cell growth pattern, and therefore, this cell line was not used in the DC experiments. The first discovery from these experiments was that either culture condition caused the new appearance of miR-375 in both MRC-5 and primary fibroblasts (Fig. 3a, c). The amount of detected miRNA varied substantially, with the largest amount under DC and the lowest under CM conditions (Fig. 3a, c). Since neither of the fibroblast lines expressed miR-375 when cultured alone, and none of the culture conditions induced pri-miR-375 (Fig. 3b, d), miR-375 expression was not induced in fibroblasts; rather, miR-375 was transferred from MCC cells to fibroblasts.

Next, we analyzed the functional effects of the EVs’ cargo transferred from MCC cells, including miR-375, on fibroblasts by measuring the mRNA expression of CAF markers, i.e., *ACTA2*, *CXCL2* and *IL1B*. Consistent with the highest miR-375 uptake, CAF markers, especially *ACTA2*, also exhibited highest expression in fibroblasts under DC conditions (Fig. 3e to h). In addition, α-SMA expression was induced at the protein level, as evidenced by immunoblotting of cell lysates and by immunofluorescence (IF) staining (Fig. 3i–j). The latter assay also revealed substantial morphological changes consistent with a CAF-like phenotype in fibroblasts, such as the development of an elongated, stellate shape, under coculture conditions (Fig. 3k, l). It was previously described that fibroblast polarization is characterized by change in the cell’s aspect ratio [41]. Indeed, quantification of the cell aspect ratio under the respective culture condition confirmed such a change which was most prominent for both MRC-5 cells and primary fibroblasts if direct cell-cell contact was possible (Fig. 3m, n).

### miR-375 alone causes fibroblast polarization

**Fig. 4:**
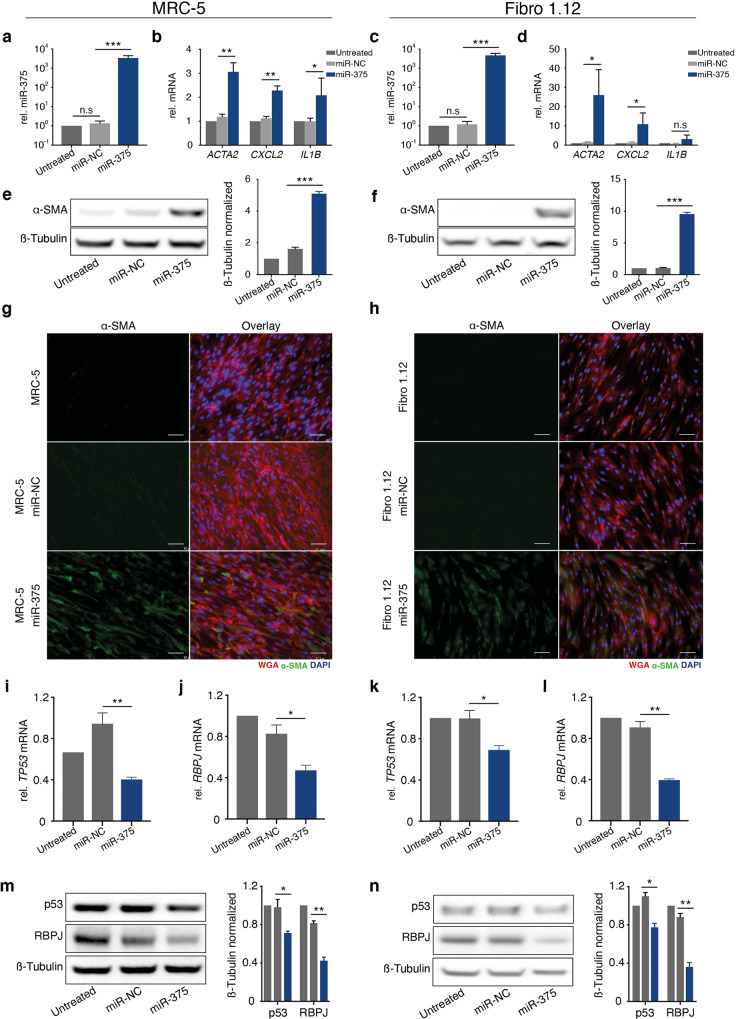
Overexpression of miR-375 in MRC-5 and primary skin fibroblasts induces a CAF-like phenotype. miR-375 was overexpressed in MRC-5 cells (**a, b, e, g, i, j, m**) or primary skin fibroblasts (Fibro1.12) (**c, d, f, h, k, l, n**). **a, c**: The relative miR-375 expression levels in untreated, miR negative control (NC)-transfected, and miR-375 mimic-transfected fibroblasts were determined by RT-qPCR. **b, d** The relative *ACTA2*, *CXCL-2* and *IL1B* mRNA expression levels in untreated, miR-NC-transfected and miR-375 mimic-transfected fibroblasts were determined by RT-qPCR. **e, f** α-SMA protein expression in untreated, miR-NC-transfected and miR-375 mimic-transfected fibroblasts was determined by immunoblotting; β-tubulin served as loading control. Quantification was performed using ImageJ. **g, h** IF staining of α-SMA (green) in untreated, miR-NC-transfected and miR-375 mimic-transfected fibroblasts. Cellular membranes were stained with WGA (red) and nuclei with DAPI (blue). Scale bars represent 50 µm. **i- l** The relative *TP53* and *RBPJ* mRNA expression levels in untreated, miR-NC-transfected and miR-375 mimic-transfected fibroblasts were determined by RT-qPCR. **m, n** p53 and RBPJ protein expression in untreated, miR-NC-transfected and miR-375 mimic-transfected fibroblasts was determined by immunoblotting; β-tubulin served as loading control. Quantification was performed using ImageJ. For RT-qPCR experiments, Cq values were normalized to U6 or *HPRT* expression and compared to the ΔCq value of untreated MRC-5 or Fibro1.4 cells, respectively. Experiments were biologically replicated trice and were performed in triplicates. The error bars indicate the SDs; * indicates *p* < 0.05, ** indicates *p* < 0.01, *** indicates *p* < 0.001.

Since many factors transferred from MCC cells may cause fibroblast polarization, we next evaluated the extent to which miR-375 causes the observed effect. Thus, we experimentally induced miR-375 in fibroblasts (Fig. 4a, c). Ectopic expression of miR-375 resulted in increased mRNA expression of *CXCL2*, *IL1B* and, predominantly, *ACTA2* (Fig. 4b, d). α-SMA protein expression was also induced upon ectopic expression of miR-375, as determined by immunoblotting (Fig. 4e, f) and IF staining (Fig. 4g, h). In addition, the morphology of these fibroblasts changed toward a CAF phenotype but to a lesser extent than in fibroblasts cocultured with miR-375-expressing MCC cells (Fig. 4g, h; Supplementary Fig. S3). Thus, these results provide conclusive evidence that miR-375 is sufficient for fibroblast polarization.

Most miRNAs function by interfering with the stability of mRNA or inhibiting its translation; *RBPJ* and *TP53* are target genes of miR-375 [26, 35] and for both reduced expression is associated with fibroblast polarization [42–44]. Here, we indeed observed significant downregulation of *RBPJ* and *TP53* mRNA and protein expression upon experimental induction of miR-375 expression (Fig. 4i–n). These observations indicate that miR-375-mediated inhibition of RBPJ and p53 is at least one of the mechanisms causing MCC-induced fibroblast polarization.

### miR-375 antagomirs attenuate MCC-induced fibroblast polarization

**Fig. 5:**
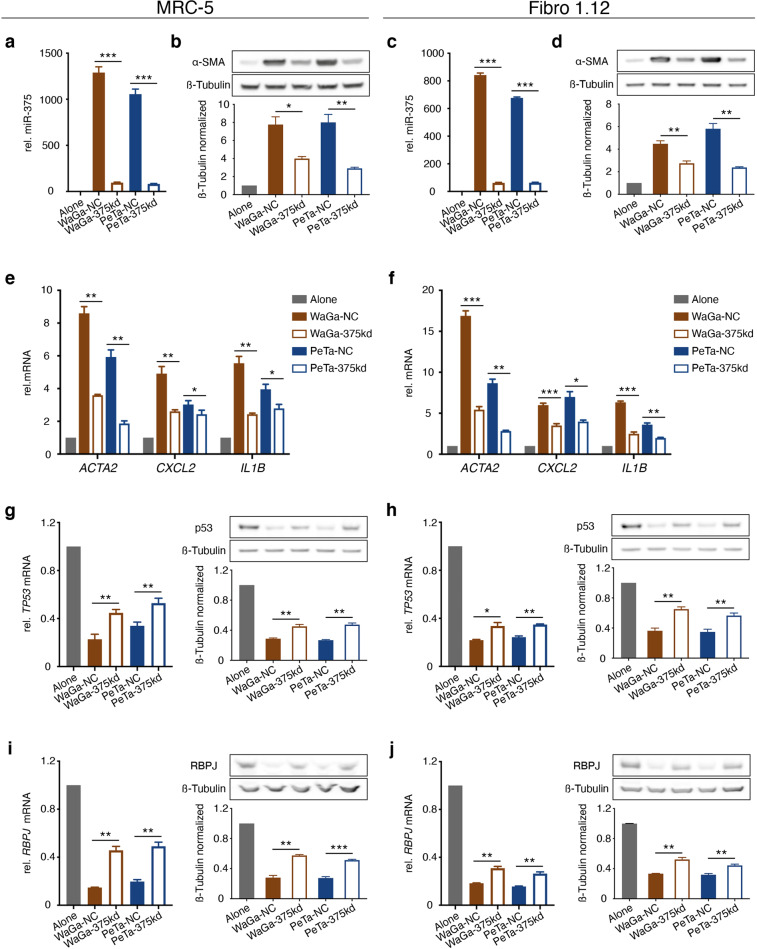
miR-375 antagomirs in MCC cells diminish coculture induced fibroblast polarization. MRC-5 cells (**a, b, e, g, i**) or Fibro 1.12 primary skin fibroblasts (**c, d, f, h, j**) were directly cocultured with WaGa or PeTa cells transfected with miR-375 antagomirs for 72 h. **a, c** The relative miR-375 expression level in fibroblasts under the indicated conditions was determined by RT-qPCR. **b, d** α-SMA protein expression in fibroblasts under the indicated conditions was determined by immunoblotting; β-tubulin served as loading control. **e, f** The relative *ACTA2*, *CXCL2* and *IL1B* expression level in fibroblasts under the indicated conditions was determined by RT-qPCR. **g, h** Relative expression of TP53 mRNA (left) and protein (right) level in fibroblasts under the indicated conditions was determined by RT-qPCR and immunoblotting. **i, j** Relative expression of RBPJ mRNA (left) and protein (right) level in fibroblasts under the indicated conditions was determined by RT-qPCR and immunoblotting. For all RT-qPCR experiments, Cq values were normalized to *HPRT* mRNA expression and compared to the ΔCq of the corresponding untreated MRC-5 or Fibro.1.12 cells transfected. Experiments were biologically replicated trice and were performed in triplicates. The error bars indicate the SDs; * indicates *p* < 0.05, ** indicates *p* < 0.01.

To confirm the relevance of miR-375 for MCC-induced fibroblast polarization, we repeated our coculture experiments after introduction of miR-375 antagomirs either into MCC cells or fibroblasts. The miR-375 antagomirs in MCC cells dramatically reduced the endogenous miR-375 expression causing an equally reduced content of miR-375 in corresponding CMs and isolated EVs (Supplementary Fig. S4a–c). Thus, introduction of miR-375 antagomirs into MCC cells, reduced their impact on transferring miR-375 to fibroblasts by either DC or TC conditions, which was associated with reduced induction of α-SMA protein and *ACTA2, CXCL2* and *IL1B* mRNA in the cocultured fibroblasts; the change in protein and mRNA expression was more pronounced under DC (Fig. 5a–f) as compared to TC conditions (Supplementary Fig. S5a–f). The observed effects of the miR-375 knockdown in MCC cells on their capacity of CAF polarization was directly explained by their reduced impact on *RBPJ* and *TP53* mRNA and protein in fibroblasts after coculture which was again more evident under DC (Fig. 5g–j) than TC conditions (Supplementary Fig. S5g–j).

**Fig. 6:**
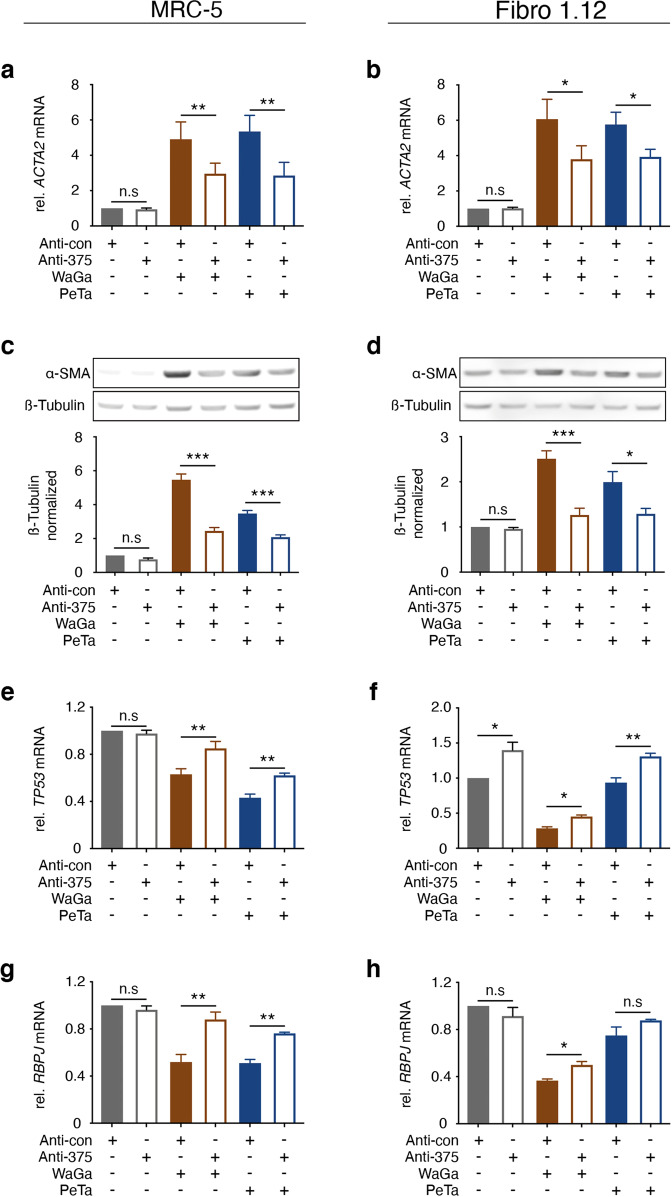
miR-375 antagomirs in fibroblasts diminish coculture induced fibroblast polarization. MRC-5 cells (**a, c, e, g**) or Fibro 1.12 primary skin fibroblasts (**b, d, f, h**) were transfected with miR-375 antagomirs or control miRNA prior to coculture with WaGa or PeTa cells in a Transwell culture system. **a, b**: The relative *ACTA2* expression level in fibroblasts under the indicated conditions was determined by RT-qPCR. **c, d**: α-SMA protein expression in fibroblasts under the indicated conditions was determined by immunoblotting; β-tubulin served as loading control. **e- h**: The relative *TP53* and *RBPJ* mRNA expression levels in fibroblasts under the indicated conditions were determined by RT-qPCR. For all RT-qPCR experiments, Cq values were normalized to *HPRT* mRNA expression and compared to the ΔCq of the corresponding untreated MRC-5 or Fibro.1.12 cells transfected with antagomir control (anta-con). Experiments were biologically replicated trice and were performed in triplicates. The error bars indicate the SDs; * indicates *p* < 0.05, ** indicates *p* < 0.01.

The introduction of the miR-375 antagomirs into fibroblasts substantially reduced the detectable amount of miR-375 transferred from MCC cells under TC or DC conditions (Supplementary Fig. S4d, e). However, the defang capacity of the antagomirs was less efficient; indeed, even in the presence of the antagomirs amounts of miR-375 detectable in fibroblasts under DC conditions exceeded the amounts observed under TC conditions in the absence of the antagomirs. Accordingly, the reversal of the coculture induced fibroblast polarization by antagomir expression in the fibroblasts was less striking (data not shown). Still, the induction of *ACTA*2 mRNA and α-SMA protein expression under TC conditions was significantly reduced in the presence of the antagomirs (Fig. 6a–d). Again, expression of *RBPJ* and *TP53* mRNA was partially rescued by the miR-375 antagomirs (Fig. 6e–h).

### Presence of miR-375 in tumor cells and CAFs from MCC tumor tissue in situ

**Fig. 7:**
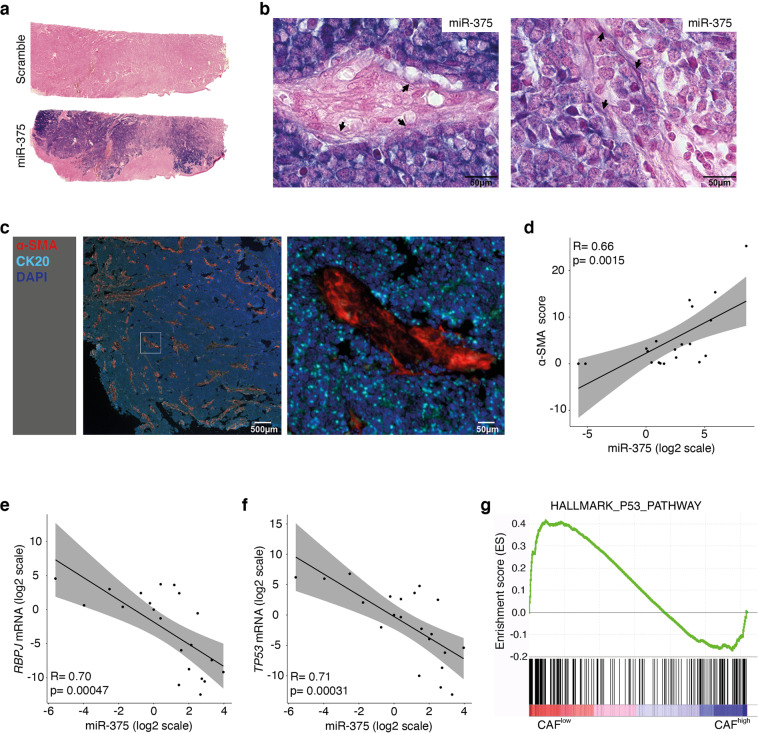
miR-375 expression correlates with fibroblast polarization in situ. **a** FFPE MCC tumor tissues were hybridized with a scrambled control probe (upper) or a miR-375-specific probe (lower). Whole slide scans of one representative MCC tissue are shown. **b** Two representative tumor areas are shown at high resolution to demonstrate the presence of miR-375 in fibroblasts (indicated by the arrows); the scale bars represent 10 µm. **c** MCC tissues stained for α-SMA and other fibroblast and MCC markers (same as Fig. 1a), and one representative MCC tissue is shown for α-SMA expression pattern. Spearman rank correlations of α-SMA staining scores (**d**), *RBPJ* (**e**) and *TP53* (**f**) mRNA expression levels with the miR-375 expression levels. Statistical analyses were performed in R using the ggpubr package. **g** GSEA was performed using scRNAseq data from two fibroblast clusters, and p53-related signaling was revealed.

To translate our in vitro observations into the clinical setting, we performed in situ hybridization (ISH) with a miR-375 probe on a series of MCC tumor samples (*n* = 6, Supplementary Table S1). miR-375 was predominantly expressed in MCC cells but was also present in stromal cells with a fibroblast morphology (Fig. 7a, b; fibroblast-like stromal cells are indicated by arrows). The miR-375-positive stromal cells were characterized by the same morphological appearance and distribution pattern as the α-SMA-expressing cells. Thus, we established a relative α-SMA staining score in 20 MCC lesions and correlated this score with miR-375 expression as determined by RT-qPCR, demonstrating a significant positive correlation (Fig. 7c, d). Furthermore, the expression of *RBPJ* and *TP53* was negatively correlated with miR-375 expression (Fig. 7e, f). Gene set enrichment analysis (GSEA) of the scRNAseq data for a primary MCC described above (Fig. 1b; Supplementary Fig. S2c) comparing the CAF^high^ and CAF^low^ fibroblast clusters based on the established CAF score demonstrated that the p53-related pathway is more strongly inhibited in CAF^high^ fibroblasts than in CAF^low^ fibroblasts (Fig. 7g). These in situ and ex vivo findings strongly support the idea that the horizontal transfer of miR-375 from MCC cells to fibroblasts triggers the polarization of the latter toward a CAF phonotype via the downregulation of RBPJ and p53.

## Discussion

In solid cancers, stromal cells have an impact at all stages of cancer progression. However, to date, fibroblasts in MCC have not been examined in detail. Here, we reveal the heterogeneity of fibroblasts in the stroma of MCC lesions, which reflects a spectrum of polarization toward a CAF phenotype. In most cancers, fibroblast polarization is triggered by tumor-derived factors such as TGF-β, PDGF, or IL-6 [6], which exhibit only low or no expression in classical MCC cell lines and tumors (data not shown). Thus, questions regarding alternative mechanisms of fibroblast polarization in MCCs are raised.

Regarding these questions, horizontal transfer of miRNAs contained within EVs has been reported to promote functional and metabolic reprogramming of fibroblasts [45, 46]. Here, we demonstrate that miR-375 transferred via EVs from MCC cells to fibroblasts targets RBPJ and p53, causing downregulation of their expression, which in turn is associated with fibroblast polarization. *RBPJ*, also called *CSL*, is a Notch mediator that suppresses CAF polarization [43, 44]. p53 downregulation allows fibroblasts to overcome cell senescence induced by RBPJ downregulation and thus enhances CAF polarization [42, 44]. Both *RBPJ* and *TP53* have been reported to be target genes of miR-375 [26, 35], and this hypothesis was confirmed by our study: Experimental expression of miR-375 reduced the expression of RBPJ and p53, which was rescued by specific antagomirs. Indeed, miR-375 expression was inversely correlated with *RBPJ* and *TP53* expression in MCC tumor tissues. Unfortunately, the mRNA expression of *RBPJ* and *TP53* in fibroblasts from MCC tumors was below the detection limit of scRNAseq. However, p53-related signaling pathways were strongly inhibited in fibroblasts with a high CAF score.

Different MCC/fibroblast coculture conditions, i.e., CM, TC, and DC, resulted in increasing effects on CAF polarization in that order. This phenomenon can be explained by the amount of miR-375 transferred to fibroblasts via EVs. In addition, direct coculture may also allow transfer through tunneling nanotubes between tumor cells and fibroblasts [47]. In addition to miR-375, other factors horizontally transferred by EVs from MCC cells to fibroblasts may induce fibroblast polarization. Secretome analysis of MCC-derived exosomes revealed more than 160 proteins [48]. Hence, importantly, ectopic expression of miR-375 alone was sufficient to polarize fibroblasts, and miR-375 antagomirs decreased phenotypic changes in fibroblasts triggered by coculture with MCC cells. Thus, our findings indicate that miR-375 plays a major role in MCC-mediated CAF polarization.

We further demonstrated the presence of miR-375 in stromal cells with morphological characteristics of fibroblasts by in situ hybridization. Quantitative differences in miR-375 levels might be a cause of CAF heterogeneity in MCC, as evidenced by the mIHC-based staining and scRNAseq results. However, different cellular origins, e.g., tissue-resident fibroblasts or recruited mesodermal stem cells, prior activation stages or additional environmental factors may contribute to the fibroblast heterogeneity in MCC [6, 49]. The importance of CAF heterogeneity in cancer progression, prognosis, and therapy has been demonstrated in many caner types. Because of their heterogeneity, there is no single exclusive marker for CAFs, but—as demonstrated here—a set of markers consisting of morphologic characteristics together with specific gene and protein expression profiles is needed to characterize this cell type. Consequently, using a single marker for isolation and/or enrichment of CAFs hold the risk of selecting of a subpopulation of cells not reflecting the complete diversity of CAFs. Although we demonstrated the uptake of EVs by fibroblasts, other stromal cells, such as lymphocytes, macrophages and endothelial cells in the microenvironment, are also likely to be affected. As reported previously, mesenchymal stromal cell-derived exosomes altered the mRNA expression and function of B lymphocytes [50] and endothelial cells [51]. Notably, transferred miR-375 enhanced tumor-associated macrophage migration and infiltration into tumor spheroids in breast cancer [52].

In summary, we established the role of miR-375 in intercellular communication between MCC tumor cells and stromal fibroblasts. We demonstrated that miR-375 derived from MCC cells or endogenously expressed miR-375 can induce fibroblast polarization in vitro and that this event probably also occurs in vivo in MCC patients. These observations suggest that miR-375 is an attractive target for therapeutic interventions; indeed, the therapeutic feasibility of targeting miRNA is currently being investigated for miR-155 in cutaneous T cell lymphoma [53].

## Material and methods

### Tumor tissues

Formalin-fixed, paraffin-embedded (FFPE) MCC tumor samples from the Department of Dermatology, University Hospital Essen were used for immunohistochemistry (IHC), IF staining and miRNA in situ hybridization. One fresh primary MCC tumor was used for scRNAseq analysis. All tumor samples were excised for diagnostic or therapeutic reasons and were confirmed to be MCC according to established histological and immune-histochemical diagnostic guidelines. The study was conducted in accordance with ethical guideline provided in the ‘Declaration of Helsinki’. All studies on human material were approved by the ethics committee of the University Duisburg-Essen (11-4715; 17-7538-BO). Informed consent was obtained for use in research.

### Multiplex immunohistochemistry (mIHC)

mIHC was performed using an Opal 7-color kit (PerkinElmer, Velbert, Germany) according to the manufacturer’s instructions. In brief, FFPE tissue sections were incubated at 60 °C for 1 h, deparaffinized in xylene and rehydrated with 100%, 96% and 70% ethanol for 2 min each. Sections were then cross-linked to slides by incubation in 10% formalin for 10 min before two 5-min washes in distilled water. Before incubation with each antibody, antigen retrieval was performed by microwaving the sections at 100 W in a suitable Opal antigen retrieval buffer with a pH of 9 (AR9) or a pH of 6 (AR6) for 15 min. After cooling at room temperature in the dark for 20 min, sections were blocked with antibody blocking solution for 10 min before incubation for 30 min at room temperature with the appropriate primary antibody (Supplementary Table S2). After three washes in TBS+ 0.05% Tween 20 (TBST), sections were incubated with Opal Polymer HRP Mouse and Rabbit reagent for 10 min at room temperature. After three washes in TBST, the appropriate Opal fluorophore was added at a dilution of 1:50 and incubated for 10 min at room temperature. After three washes in TBST, the process was repeated for reaction with the next antibody. The order in which the antibodies were added to the sections is summarized in Supplementary Table S2. After the final antibody incubation, nuclei were stained with 4’,6-diamidino-2-phenylindole (DAPI) for 5 min, and sections were embedded in ProLong Diamond Antifade Mountant (Thermo Fisher Scientific, Oberhausen, Germany) and incubated at room temperature in the dark for 24 h before imaging with a Mantra quantitative pathology workstation (PerkinElmer).

### Multiplex scRNAseq and data analysis

Tissue sections of in total three samples (MCC_1, MCC_2, MCC_3) were dissociated into single-cell suspensions and barcoded using the 10x Genomics Chromium v2.0 platform (10x Genomics, Leiden, Netherland) [54]. Library preparation was performed according to the 10x Genomics protocol, and the library was sequenced on an Illumina HiSeq 4000 platform (Illumina, Eindhoven, Netherland). Cell Ranger Single-Cell Software Suite version 2.1.1 (http://10xgenomics.com/) was used to align cDNA reads to the hg19 human reference genome. Cell types were annotated using marker genes (proliferating cells: MKI67 and TOP2A, fibroblasts: VIM, FN1, and S100A4; T cells: CD3E and CD8A; MCC cells: CHGA, ENO2, KRT20, and CD44; and endothelial cells: VIM, VWF, and CAV1). Afterwards, fibroblasts of all three samples were extracted and other cell types discarded. In total, we found 71 single fibroblast cells in MCC#1, 610 in MCC#2 and 120 in MCC#3. Normalized and raw expression counts for the fibroblast single cell analysis were uploaded to Figshare (https://doi.org/10.6084/m9.figshare.13095293), together with the respective cell meta data. This includes the CAF signature score which was computed using the Seurat AddModuleScore function and based on a list of 88 CAF-associated genes published by Tirosh et al. [38] (Supplementary Table S3). For normalization, dimension reduction, clustering, and visualization, we used Seurat R package v2 [55]. In order to correct for batch effects between the samples we applied the harmony package and adjusted the PCs calculated with Seurat [56]. Differentially expressed genes were called using Seurat’s FindMarkers function with the default settings. RNA velocity estimates were calculated using the corresponding velocyto R package with a gene-relative model based on PCA, and cell-cell distances calculated by Seurat [39]. Extrapolated cell states were projected onto PCA plot with the speed vectors that summarizes the transcriptional variability in the data in a lower-dimensional space using the “pca.velocity.plot“ function. Inferred trajectories are depicted as arrows, with the base of the arrow indicating the current cell state and the tip of the arrow indicating the extrapolated cell state.

### Cell culture

All MCC cell lines, the cutaneous squamous cell carcinoma cell line SCL-2 [57] and the fibroblast cell line MRC-5, along with their culture conditions and growth patterns, have been described before [27]. Primary skin fibroblasts were isolated and cultured in our laboratory. All cell lines are regularly authenticated by short tandem repeat analysis (last performed in June 2019) and mycoplama test was performed monthly in our lab.

CM was generated by culturing cells (2 × 10^6^) in fresh RPMI 1640 medium supplemented with 10% fetal bovine serum (FBS, PAN-Biotech, Aidenbach, Germany) and 1% penicillin/streptomycin (P/S, PAN-Biotech) for 72 h. Culture supernatants were centrifuged to remove floating cells and cell debris. CM was added to 70% confluent fibroblasts and incubated for 72 h.

For Transwell chamber system coculture (TC), 5×10^5^ MRC-5 cells or 2 × 10^5^ primary skin fibroblasts were seeded in the lower chambers of a 6-well plate with 0.4 µm pore size polyester membrane inserts (Corning, Hagen, Germany). After 24 h, 1 × 10^6^ MCC cells were seeded in the inserts. Fibroblasts were harvested after 72 h of coculture.

For direct coculture (DC), MCC cells were added to 70% confluent, adherent, growing fibroblasts. After 72 h of coculture, the supernatants and floating cells were carefully removed, and the remaining adherent cells were washed at least 3 times with PBS to remove all floating cells. Adherent fibroblasts were harvested.

### Generation of primary skin fibroblasts

Fibro1.4 and Fibro1.12 primary skin fibroblasts were generated from biopsies of healthy skin. Biopsies were cut into small pieces and incubated with 130 µM dispase II (Sigma-Aldrich, Darmstadt, Germany) in Hank’s balanced salt solution at 4 °C overnight to separate the dermis from the epidermis. The dermis was then transferred into fresh medium 1:1 DMEM + DMEM/F-12 (PAN-Biotech) supplemented with 1% P/S, 15% FBS, 10 µM amphotericin B (AMP-B, Sigma-Aldrich) and 75 µM ciprofloxacin (CIPRO, Sigma-Aldrich) and incubated overnight in a humidified incubator at 37 °C. The medium was replaced after 24 h and 48 h, gradually reducing the AMP-B and CIPRO concentrations to 5 µM and 35 µM, respectively. Then, the medium was changed weekly until fibroblasts began to form confluent patches. At this point, the excess tissues were discarded, and the remaining adherent fibroblasts were cultured in 1:1 DMEM + DMEM/F-12 supplemented with 1% P/S and 15% FBS. The pcDH_EGFP_puro plasmid and helper plasmids (pHCMV-G, pRSV rev and pMDLg/pRRE) were used to generate EGFP-labeled fibroblasts. Lentiviral supernatants were produced from HEK293T cells two days following transfection, and virus-containing supernatants were harvested and filtered through 0.45 μm pore size filters. Polybrene was added (5 μg/ml) for infection. After 16 h of incubation, the target cells were washed twice with culture medium and subjected to puromycin selection (1 μg/ml).

### EVs isolation and characterization

EVs were isolated from 2 ml of CM using an ExoQuick-TC kit (System Biosciences, Palo Alto, CA, USA) following the manufacturer’s instructions. Isolated fractions of EVs were analysed in the violet side scatter channel with a CytoFLEX flow cytometer (Beckman Coulter, Brea, California, USA). Standardized nanoparticles of 50 nm, 100 nm and 200 nm were used to determine the sizes of the isolated EVs. The isolated EVs were stained with Exo-Red RNA fluorescent dye (System Biosciences) according to the manufacturer’s instructions.

### miRNA overexpression

MRC-5 and Fibro1.12 cells were transfected with miR-375 mimics (Mission microRNA Mimic, HMI0537, Sigma-Aldrich) or negative control miRNA (Mission miRNA negative control 1, HMC0002, Sigma-Aldrich). Lipofectamine 3000 (Thermo Fisher Scientific) was used as the transfection reagent. The final miRNA mimic concentration used for transfection was 20 nM, and transfection was performed according to the protocol provided by the manufacturer.

### miRNA in situ hybridization

Locked nucleic acid-based miRNA in situ hybridization was performed on FFPE MCC tissue sections by Bioneer in situ hybridization services (Hørsholm, Denmark). MCC sections were incubated with a specific probe against miR-375 (20 nM; DIG-TCACGCGAGCC GAACGAAA-DIG) or a scrambled control probe (20 nM; DIG-TGTA ACACGTCTATACGC CCA-DIG) at 58 °C in accordance with a previously described protocol [58].

### RT-qPCR

Total RNA from cell lines and tumor tissues was isolated using a PeqGOLD total RNA Kit (VWR/Peqlab, Erlangen, Germany) and transcribed to cDNA using SuperScript IV reverse transcriptase (Thermo Fisher Scientific) according to the manufacturer’s instructions. To isolate RNAs from cell free supernatant/EVs, the miRNeasy Mini Kit (Qiagen, Hilden, Germany) was used according to the manufacturer’s instructions; cel-miR-39 (ID000200) served as spike-in control. RT-qPCR was performed in a CFX Real-Time PCR system (Bio-Rad Laboratories, Düsseldorf, Germany). HPRT was used as the endogenous control, expression was detected using the SYBR Green method, and relative expression levels were calculated by the 2^-ΔΔCq^ method using the corresponding untreated control or as otherwise indicated. The primer sequences are listed in Supplementary Table S4.

For miRNA analysis, Applied Biosystems TaqMan MicroRNA Assays (Thermo Fisher Scientific) were performed according to the manufacturer’s instructions. Pre-designed TaqMan microRNA assays for miR-182 (ID002334), miR-106b (ID000442), miR-19b (ID000396), miR-200c (ID002300), and miR-375 (ID000564) were used. The quantification cycle threshold (Cq) values of the analyzed miRNAs were normalized to the expression of the small nucleolar RNA RNU6B (ID001093) for cellular miRNAs or spike-in cel-miR-39 (ID000200) for miRNAs in cell free supernatant/EVs; the expression relative to the corresponding comparator was calculated using the 2^-ΔΔCq^ method.

### Immunoblot analysis

Cell lysis and protein extraction were performed using RIPA buffer (Sigma-Aldrich) supplemented with complete Mini protease inhibitor cocktail (Sigma-Aldrich). Proteins were separated by SDS-PAGE on 4–12% precast gradient gels (Thermo Fisher Scientific) and transferred to nitrocellulose membranes using an iBlot dry blotting system (Thermo Fisher Scientific). After blocking with 3% milk, membranes were incubated overnight at 4 °C with specific primary antibodies (Supplementary Table S5) After three washes, the appropriate peroxidase-conjugated secondary antibodies were added (Dako), and immunoreactions were visualized using ECL Western Blotting Substrate (Pierce, Rockford, USA) in a chemiluminescence imager (Amersham Imager 600, GE Healthcare, New York, USA). Quantification of immunoblot data was performed with ImageJ and GraphPad software (GraphPad Software Inc., San Diego, CA, USA).

### IF staining

Fibroblasts were cultured on cover slips (Thermo Fisher Scientific) in 12-well plates. Before staining, cells were fixed in 4% paraformaldehyde for 10 min, permeabilized with PBS and 0.2% Tween 20 for 15 min and blocked with 1% BSA in PBS for 1 h. Then, a primary antibody specific for α-SMA (clone 1A4, Dako), diluted 1:200 in PBS containing 1% BSA, was added and incubated overnight at 4 °C. After two washes, an Alexa Fluor 546-labeled goat-anti-mouse secondary antibody (1:200, Thermo Fisher Scientific) was added for one hour. After two washes, cells were incubated with 5 µg/ml wheat germ agglutinin (WGA, conjugated to Alexa Fluor 647, Thermo Fisher Scientific) for 30 min, and DAPI (1:2000, Thermo Fisher Scientific) was added to visualize nuclei. After three washes, cells were embedded in ProLong Diamond Antifade Mountant (Thermo Fisher Scientific) and then evaluated with an Axio Observer.Z1 microscope (Zeiss, Oberkochen, Germany).

### Immunohistochemical staining

Immunohistochemical staining was performed on FFPE MCC tissue sections after deparaffinization. Antigen retrieval was performed using EDTA retrieval buffer (Sigma-Aldrich, pH 9) in a steamer at 95 °C for 20 min. After cooling for 20 min, samples were washed twice with PBS and incubated for 10 min with peroxidase blocking solution (3% H_2_O_2_ in methanol) at room temperature. Sections were then incubated for 30 min at room temperature with an antibody specific for α-SMA (clone 1A4, Dako) diluted 1:100 in PBS containing 3% BSA. After incubation with a horseradish peroxidase (HRP)-polymer anti-mouse antibody (POL2DS-006, Zytomed, Eching, Germany), detection was performed using Permanent HRP Green stain (Zytomed). After nuclei were counterstained with haematoxylin, sections were dehydrated and mounted in Vitro-Clud mounting medium (R. Langenbrinck, Emmendingen, Germany). The stained sections were then scored for α-SMA-positive fibroblasts using the Mantra quantitative pathology workstation with the inForm analysis software (PerkinElmer). In detail, the instrument was trained by manually categorizing the tissue sections into tumor and stromal segments, followed by automated cell segmentation via haematoxylin-stained nuclei. The system was then trained to automatically distinguish between cell phenotypes by manually identifying 100 tumor and 100 stromal cells. The final α-SMA staining score was calculated as the percentage of α-SMA-positive stromal cells. The cut-off for α-SMA-positive cells was set at an optical intensity intensity of greater than 0.26. For each section, four areas at the border of the tumor and four areas in the middle of the tumor were selected and analyzed as explained above. The means of the values for the tumor border and of those for the tumor middle were calculated for the final scoring.

### GSEA

GSEA version 3.0 was used to conduct GSEA [59]. Pre-ranked analyses were performed by sorting differentially expressed genes according to their fold changes without prior filtering on significance or effect size. Enrichment was then tested with the hallmarks of cancer gene set [60].

### Statistical analysis

Statistical analyses were performed using GraphPad Prism 8.0 (GraphPad Software Inc.) Experiments containing two groups of data were analyzed using the Mann–Whitney U test. Experiments containing more than two groups of data were analyzed using the Kruskal-Wallis test for unpaired nonparametric samples. Correlation analyses were performed in R Studio using the ggpubr R package. A *p* value < 0.05 was considered significant; the corresponding *p* values are indicated in the figures as follows: *p < 0.05, **p < 0.01, and ***p < 0.001.

## Supplementary information

Supplementary figures

Supplementary tables
