# Supplementary material for: Merkel cell carcinoma-derived exosome-shuttle miR-375 induces fibroblast polarization by inhibition of RBPJ and p53
Source: Oncogene. 2020 Dec 11;40(5):980–96. doi: 10.1038/s41388-020-01576-6 (PMC7862059; doi:10.1038/s41388-020-01576-6)
Supplement: Supplementary file 1 — Supplementary figures [file 41388_2020_1576_MOESM1_ESM.pdf]

Suppl. Figure S1

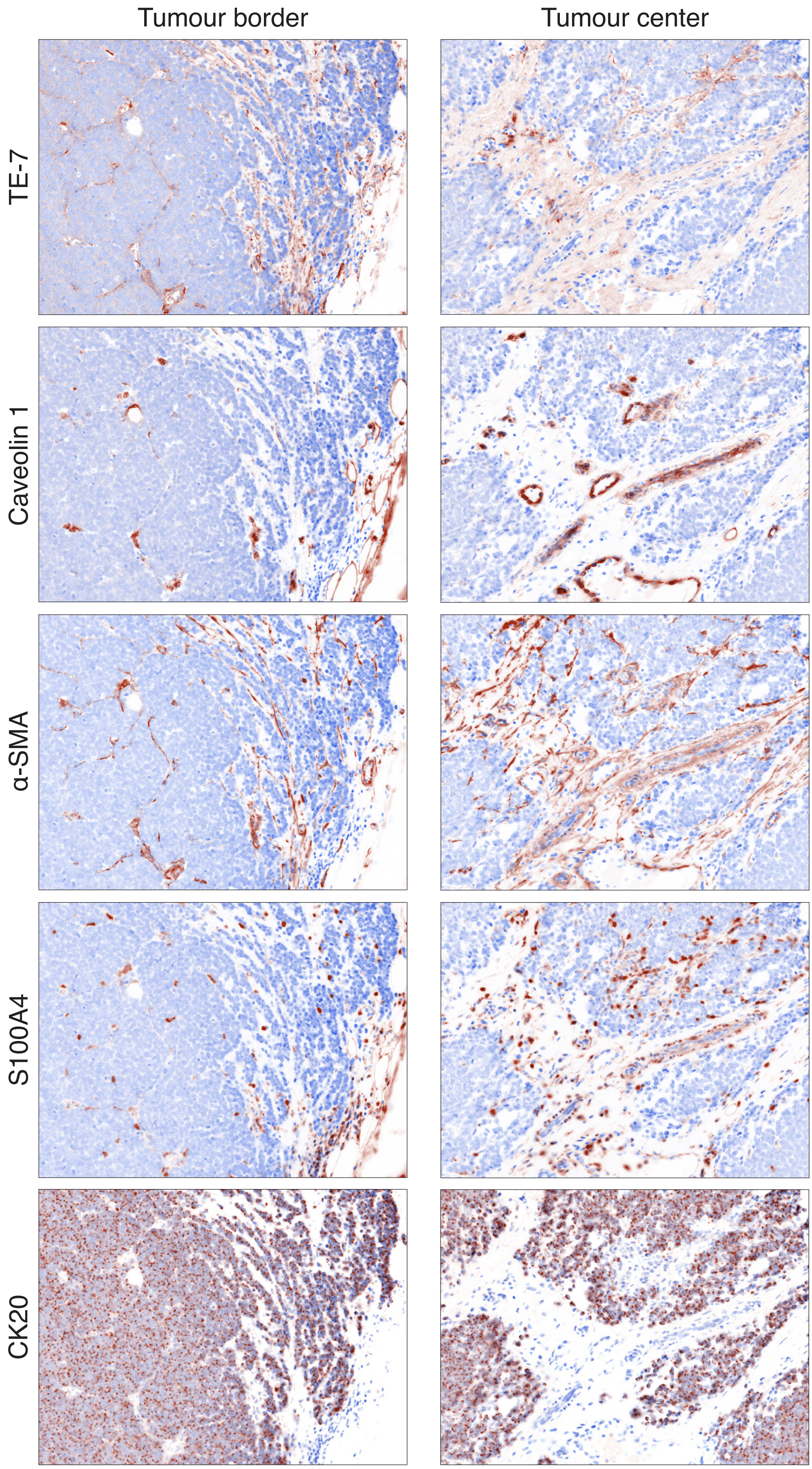

Suppl. Fig. S1: Expression of fibroblast activation markers in MCC via Pathology Views
Pathology Views™ was applied to generate six single marker pseudo-bright field images of the images shown in Fig. 1a in which the individual signals for TE-7, Caveolin-1, α-SMA, S100A4 and CK20 were converted into 3, 3 diaminobenzidine (DAB)-like color. of the estimated future cell state of each cell.

Suppl. Figure S2

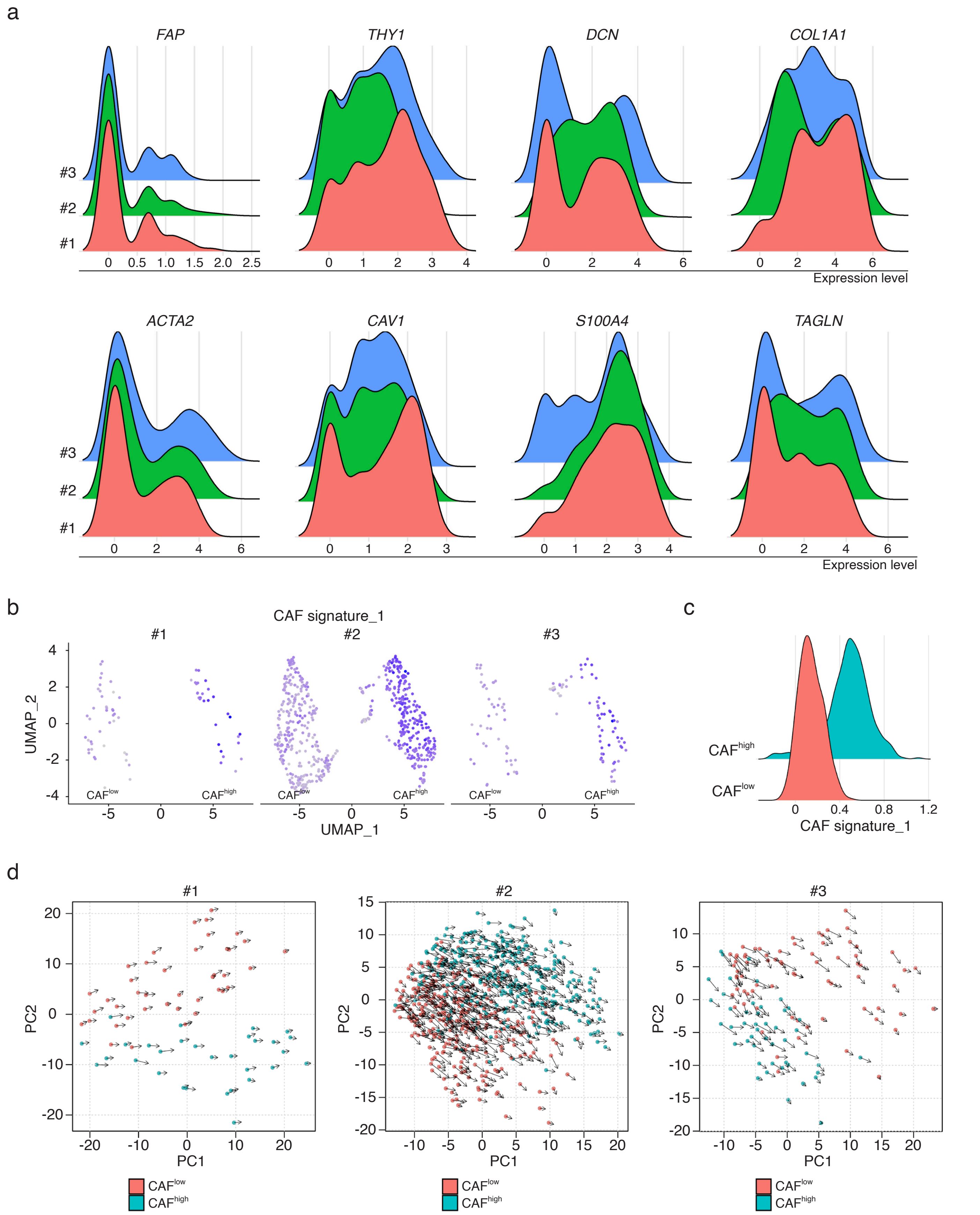

Suppl. Fig. S2: Heterogeneity of the mRNA expression of fibroblast polarization-related genes in three MCC tumors a: mRNA expression levels of selected CAF markers in identified fibroblasts in three MCC tumors. b: UMAP plot of the CAF signature scores of fibroblasts in three tumors. These fibroblast clusters were annotated as CAFhigh and CA-Flow. c: CAF signature scores were generated for merged fibroblast clusters from three tumors based on the mRNA expression of 88 CAF marker genes. d: PCA plot of the RNA velocity with the speed vectors of the two fibroblast clus-ters in three tumors. The arrows indicate the location of the estimated future cell state of each cell.

Suppl. Figure S3

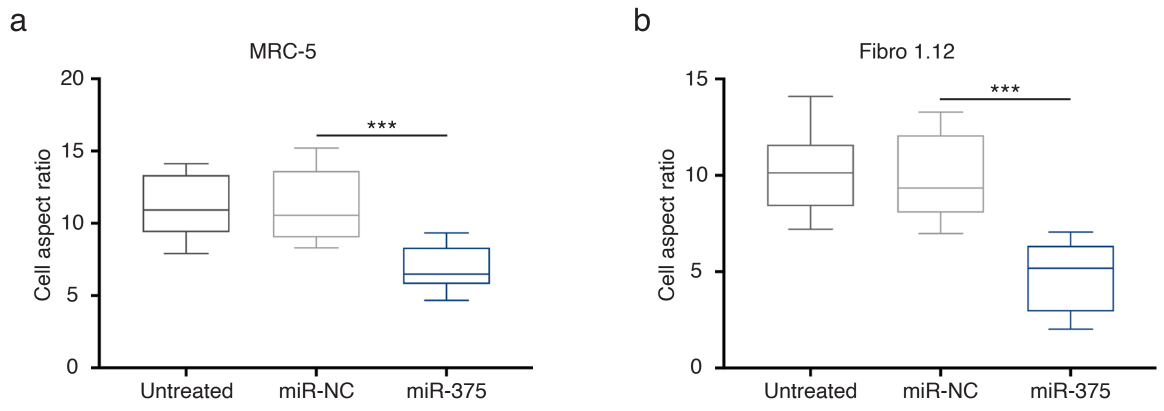

Suppl. Fig. S3: Overexpression of miR-375 in MRC-5 and primary skin fibroblasts induces a CAF-like morphology change

a- b: The graphs show the values of cell aspect ratio (length/ width) of MRC-5 (a) and Fibro1.12 (b) under indicated conditions (n= 50), presented as box-and-whisker plots. *** indicates $p<0.001$.

Suppl. Figure S4

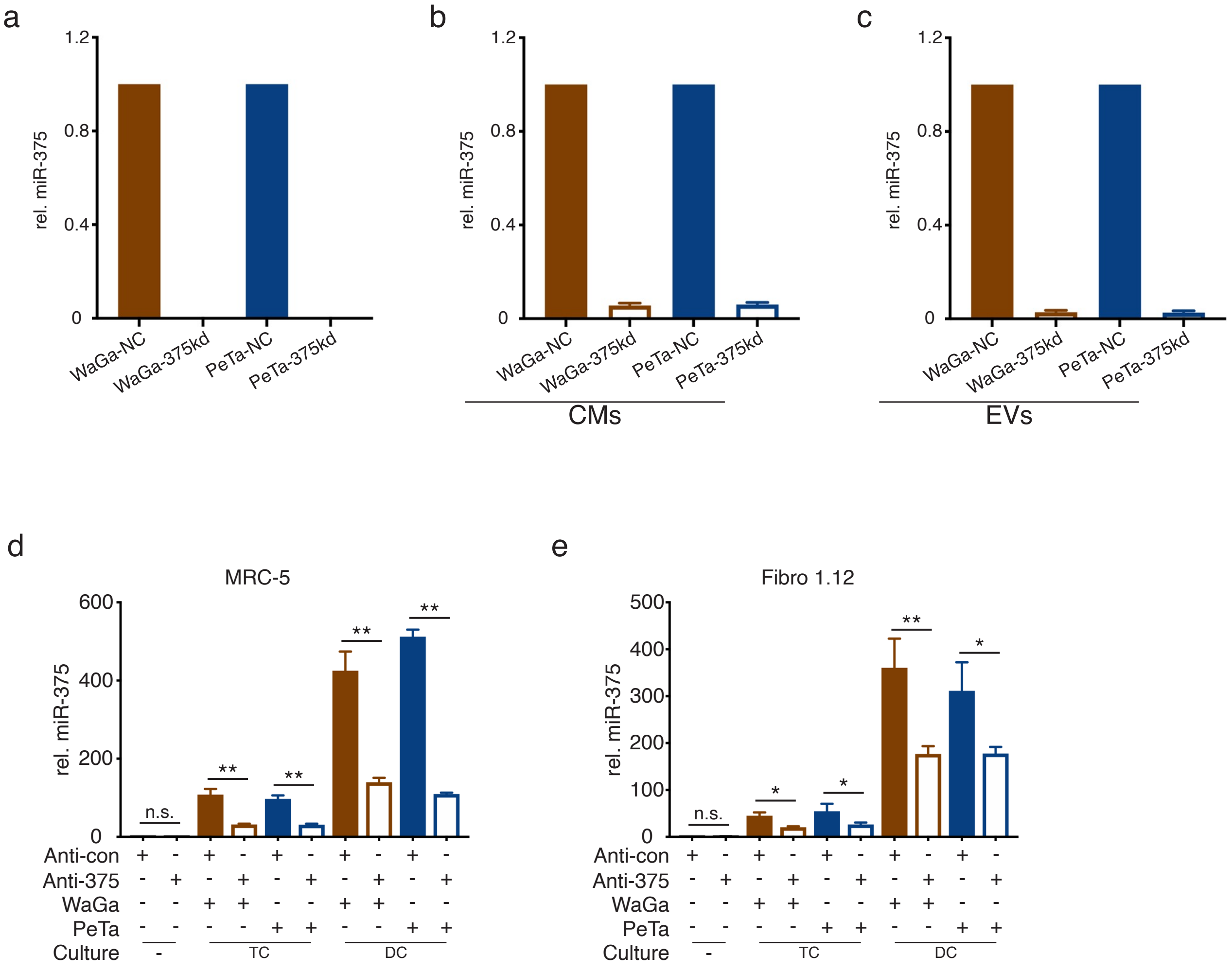

Suppl. Fig. S4: miR-375 antagomirs transfected into MCC cells or fibroblasts result in miR-375 knockdown
a- c: miR-375 antagomirs were transfected into WaGa and PeTa cells via nucleofection. Total RNAs from MCC cells, conditioned mediums (CMs) and isolated Extracellular vesicles (EVs) were used for miR-375 expression determination. The relative miR-375 expression level in MCC cells (a), CMs (b) and EVs (c) under the indicated conditions was determined by RT-qPCR. d, e: miR-375 antagomirs were transfected into MRC-5 and Fibro 1.12 cells using lipofectamine. The relative miR-375 expression level in fibroblasts under the indicated coculture conditions was determined by RT-qPCR. Cq values were normalized to U6 expression (a, d, e) or spiked cel-miR-39 (b, c) and compared to the ΔCq of the corresponding control. Experiments were biologically replicated trice and were performed in triplicates. The error bars indicate the SDs; * indicates $p<0.05$, ** indicates $p<0.01$.

Suppl. Figure S5

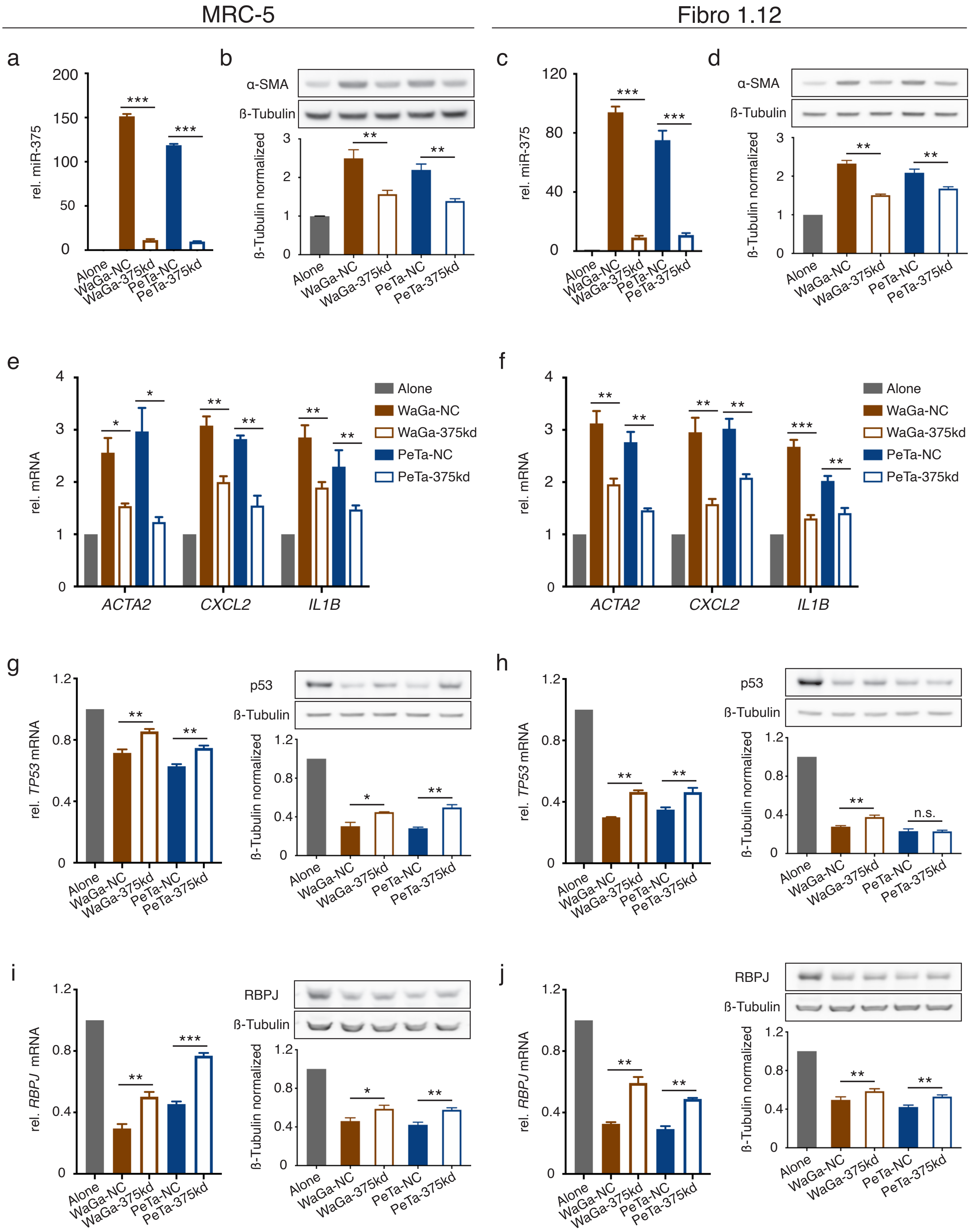

Suppl. Fig. S5: miR-375 antagomirs in MCC cells diminish co-culture induced fibroblast polarization
MRC-5 cells (a, b, e, g, i) or Fibro 1.12 primary skin fibroblasts (c, d, f, h, j) were co-cultured with WaGa or PeTa cells transfected with miR-375 antagomirs in Transwell system for 72 hours. a, c: The relative miR-375 expression level in fibroblasts under the indicated conditions was determined by RT-qPCR. b, d: α-SMA protein expression in fibroblasts under the indicated conditions was determined by immunoblotting; β-tubulin served as loading control. e, f: The relative ACTA2, CXCL2 and IL1B expression level in fibroblasts under the indicated conditions was determined by RT-qPCR. g, h: Relative expression of TP53 mRNA (left) and protein (right) level in fibroblasts under the indicated conditions was determined by RT-qPCR and immunoblotting. i, j: Relative expression of RBPJ mRNA (left) and protein (right) level in fibroblasts under the indicated conditions was determined by RT-qPCR and immunoblotting. For all RT-qPCR experiments, Cq values were normalized to HPRT mRNA or U6 expression and compared to the ΔCq of the corresponding untreated MRC-5 or Fibro.1.12 cells. Experiments were biologically replicated trice and were performed in triplicates. The error bars indicate the SDs; * indicates $p<0.05$, ** indicates $p<0.01$, *** indicate $p< 0.001$.
