# Supplementary material for: Merkel cell carcinoma-derived exosome-shuttle miR-375 induces fibroblast polarization by inhibition of RBPJ and p53
Source: Oncogene. 2020 Dec 11;40(5):980–96. doi: 10.1038/s41388-020-01576-6 (PMC7862059; doi:10.1038/s41388-020-01576-6)
Supplement: Supplementary file 2 — Supplementary tables [file 41388_2020_1576_MOESM2_ESM.docx]

**Supplementary Tables**

*Table S1: MCPyV status of analyzed tumors*

| Tumor Nr. | MCPyV status | Used in scRNAseq | Used in mIHC | Used miRNA in situ hybridization |
| --- | --- | --- | --- | --- |
| #1 | negative | Yes, Fig. 1b, c & Suppl. Fig. 2 | Yes, Fig. 1a & 7c | Yes, Fig. 7a, b |
| #2 | negative | Yes, Fig. 1b, c & Suppl. Fig. 2 | Yes | Yes |
| #3 | negative | Yes, Fig. 1b, c & Suppl. Fig. 2 | Yes |  |
| #4 | positive |  | Yes | Yes |
| #5 | positive |  | Yes | Yes |
| #6 | positive |  | Yes | Yes |
| #7 | positive |  | Yes | Yes |
| #8 | positive |  | Yes |  |
| #9 | negative |  | Yes |  |
| #10 | positive |  | Yes |  |

*Table S2: Primary antibodies used for Multicolor Immunofluorescence(mIHC)*

| # | Antigen retrieval | Antibody | Clone number | Clonality | Supplier | Dilution | Fluorophore |
| --- | --- | --- | --- | --- | --- | --- | --- |
| 1 | AR9 | Anti-fibroblast | TE-7 | monoclonal | Millipore | 1:600 | Opal 520 |
| 2 | AR9 | Caveolin 1 | PA5-17447 | polyclonal | Thermo Fischer | 1:500 | Opal 570 |
| 3 | AR9 | 𝑎-SMA | 1A4 | monoclonal | Dako | 1:600 | Opal 540 |
| 4 | AR9 | S100A4 | EPR2761(2) | monoclonal | Abcam | 1:1200 | Opal 620 |
| 5 | AR6 | FAP | HPA059739 | polyclonal | Atlas antibody | 1:500 | Opal 650 |
| 6 | AR6 | CK20 | D9Z1Z | monoclonal | Cell signaling | 1:800 | Opal 690 |

*Table S3: CAF-associated genes used for CAF signature 1*

| FAP | MFAP5 | IGF2 | C1R | MXRA8 | PCSK5 |
| --- | --- | --- | --- | --- | --- |
| THY1 | MEG3 | GFPT2 | ANTXR1 | PDGFRL | ELN |
| DCN | SULF1 | PDGFRA | MGST1 | COL14A1 | CXCL12 |
| COL1A1 | AOX1 | CRISPLD2 | C3 | RCN3 | OLFML2B |
| COL1A2 | SVEP1 | CPE | PALLD | LTBP2 | PLAC9 |
| COL6A1 | LPAR1 | F3 | FBN1 | NID2 | FHL2 |
| COL6A2 | PDGFRB | MFAP4 | CPXM1 | ADAM33 | CTGF |
| COL6A3 | TAGLN | C1S | CYBRD1 | PRRX1 | RARRES2 |
| CXCL14 | IGFBP6 | PTGIS | IGFBP5 | PCOLCE |  |
| LUM | FBLN1 | LOX | PRELP | SCARA3 |  |
| COL3A1 | CA12 | CYP1B1 | PAPSS2 | AMOTL2 |  |
| DPT | SPOCK1 | CLDN11 | MMP2 | TPST1 |  |
| ISLR | TPM2 | SERPINF1 | CKAP4 | MSC |  |
| PODN | THBS2 | OLFML3 | CCDC80 | VASN |  |

*Table S4: Primers used in qRT-PCR*

| Gene | Forward primer | Reverse primer |
| --- | --- | --- |
| *ACTA2* | CAGCCAAGCACTGTCAGG | CCAGAGCCATTGTCACACAC |
| *CXCL2* | GGGCAGAAAGCTTGTCTCAA | GCTTCCTTCCTTCTGGT |
| *IL1B* | GAGCTGATGGCCCTAAACA | AAGCCCTTGCTGTAGTGGTG |
| *TGFB1* | CGCCAGAGTGGTTATCT | TAGTGAACCCGTTGATGTC |
| *CXCR4* | GTTAATGCTTGCTGAATTGGAA | CTCGGTGTAGTTATCTGAAGTG |
| *HPRT* | GTCGTGATTAGTGATGATG | GTTCAGTCCTGTCCATAA |
| Pri-miR-375 | CCTCACCTGAACGCATCTG | TGGGGACGAAGCCAAGCTA |
| *RBPJ* | CGGCCTCCACCTAAACGAC | TCCATCCACTGCCCATAAGAT |
| *TP53* | CAGCACATGACGGAGGTTGT | TCATCCAAATACTCCACACGC |

*Table S5: Primary antibodies used for Immunoblot*

| # | Antibody | Clone number | Supplier | Dilution |
| --- | --- | --- | --- | --- |
| 1 | CD63 | H5C6 | Biosciences | 1:500 |
| 2 | Tsg101 | 4A10 | Thermo Fisher Scientific | 1:1000 |
| 3 | Calnexin | 2433 | Cell Signaling Technology | 1:1000 |
| 4 | 𝑎-SMA | 1A4 | Dako | 1:1000 |
| 5 | p53 | DO7 | Dako | 1:500 |
| 6 | RBPJ | D10A4 | Cell Signaling Technology | 1:1000 |
| 7 | β-tubulin | TUB2.1 | Sigma-Aldrich | 1:2000 |
